# Virtual Reality for Anxiety Reduction: A Mixed‐Methods Preliminary Study on Blue Space Environments Among Healthy Participants

**DOI:** 10.1155/da/3317133

**Published:** 2026-07-15

**Authors:** Adisti Dwijayanti, Aida Rosita Tantri, Dina Muktiarti, Imelda Rosalyn Sianipar, Uti Nilam Sari, Natalia Widiasih Raharjanti, Rima Maulida Hidayati, Minhua Ma

**Affiliations:** ^1^ Department of Medical Pharmacy, Faculty of Medicine, Universitas Indonesia, Jakarta, Indonesia, ui.ac.id; ^2^ Simulation Based Medical Education and Research Cluster (SIMUBEAR), Indonesian Medical Education and Research Institute (IMERI), Jakarta, Indonesia; ^3^ Department of Anesthesiology and Intensive Care, Faculty of Medicine, Universitas Indonesia, Jakarta, Indonesia, ui.ac.id; ^4^ Department of Child Health, Faculty of Medicine, Universitas Indonesia, Jakarta, Indonesia, ui.ac.id; ^5^ Department of Medical Physiology and Biophysics, Faculty of Medicine, Universitas Indonesia, Jakarta, Indonesia, ui.ac.id; ^6^ Teknologi Informasi Medimedi, Depok, Indonesia; ^7^ Department of Psychiatry, Faculty of Medicine, Universitas Indonesia, Jakarta, Indonesia, ui.ac.id; ^8^ Department of Psychiatry, University of Oxford, Oxford, UK, ox.ac.uk

**Keywords:** anxiety reduction, blue space, cybersickness, user experience, virtual reality

## Abstract

**Background:**

Anxiety is a prevalent issue among cancer patients undergoing chemotherapy, significantly impacting their treatment and quality of life. Virtual reality (VR) has emerged as a promising nonpharmacological intervention to reduce anxiety by providing immersive, distraction‐based experiences. This study introduces a VR application featuring the Seribu Islands, designed to alleviate anxiety through exposure to natural environments.

**Objective:**

To assess the effectiveness of the VR Seribu Island in reducing anxiety, evaluate user experience (UX), and identify potential cybersickness symptoms among healthy participants.

**Methods:**

A mixed‐methods design was used to evaluate 30 healthy participants who interacted with the VR Seribu Island. Pre and Postexposure anxiety levels were measured using the State–Trait Anxiety Inventory (STAI). UX was assessed via a questionnaire, and semi‐structured interviews explored participant perceptions. Cybersickness symptoms were evaluated using the Cybersickness Syndrome Questionnaire (CSQ‐VR).

**Results:**

Post‐VR anxiety scores were significantly lower than pre‐VR scores (median difference = 8.0, 95% CI [4.39, 8.61], *p* = 0.001, *r* = 0.83), indicating a reduction in anxiety. Participants reported high satisfaction with the VR experience, with an average score of 4.48 out of 5. Cybersickness symptoms were minimal, with low severity reported across most metrics.

**Conclusions:**

The VR Seribu Island significantly reduced anxiety and was well received by participants. This preliminary study supports further investigation of VR as a therapeutic tool for anxiety management in clinical populations.

## 1. Introduction

Cancer is one of the noncommunicable diseases whose prevalence of new cases continues to increase and is the leading cause of death in the world [1]. Based on data from the International Agency for Research on Cancer (IARC), it is estimated that there will be around 29.5 million new cases and 16.5 million deaths due to cancer in 2040 [2]. In Indonesia, there were 396,914 new cases and 234,511 deaths due to cancer in 2020 [3]. Indonesia ranks 8th in Southeast Asia and 23rd in Asia with a cancer incidence of 136.2 per 100,000 population [4].

Cancer significantly affects the daily lives of sufferers. Long‐term treatment such as chemotherapy has effects or disorders on the patient’s physical condition, including nausea and vomiting, anorexia, sleep disorders, and pain [5]. In addition to the emergence of physical disorders, it also causes psychological problems such as anxiety disorders and depression [6]. Anxiety in cancer patients can arise from various factors such as reactions to a cancer diagnosis, severe pain, long‐term treatment, side effects of treatment, and feelings of being burdened or dependent on others [7]. Around 55% of cancer patients experience symptoms of anxiety, which increases to 77% in patients undergoing chemotherapy [8].

Anxiety has a negative effect on the treatment of cancer patients; namely, it can prolong the treatment period and reduce the quality of life if not handled properly [9]. Anxiety management in cancer patients must be carried out immediately after the patient is diagnosed with cancer and continued until treatment is complete [10]. Reducing anxiety levels in cancer patients can increase positive interactions with family and the surrounding environment and can improve social, cognitive, and emotional functions in cancer patients [11].

Anxiety management in cancer patients can be done pharmacologically or nonpharmacologically. The use of pharmacological therapy with long‐term drugs such as benzodiazepines to overcome anxiety in cancer patients will cause unwanted side effects such as drug dependence, so nonpharmacological interventions are safer and are recommended [12]. There are several nonpharmacological methods that have been previously studied to reduce anxiety in cancer patients, such as deep breathing techniques, relaxation techniques, guided imagery techniques, and distraction techniques [13]. Among several choices of nonpharmacological methods to overcome anxiety in cancer patients undergoing chemotherapy, distraction techniques are one of the best choices. Distraction techniques are effective coping strategies that focus on emotions because they divert attention from unpleasant to pleasant or enjoyable stimuli, thereby reducing stress and anxiety [14].

The benefits of blue spaces in reducing stress and anxiety have been well documented [15]. Exposure to blue spaces, such as coastal areas, rivers, and lakes, has been shown to lower levels of cortisol, a key stress hormone, and enhance feelings of calm and well‐being. These effects are thought to be due to the soothing qualities of water, as well as the sensory immersion provided by the sounds, visuals, and textures unique to aquatic settings. Research indicates that time spent in blue spaces can improve mood and decrease physiological markers of stress, making them particularly valuable in therapeutic applications for stress management and mental health support in various populations, including cancer patients.

Virtual reality (VR) technology is a distraction technique that allows users to interact with a three‐dimensional (3D) environment digitally generated by a computer. The power of VR distraction lies in its ability to activate several user senses simultaneously by providing visual images, sounds, and even certain aromas/smells [16]. VR can visually isolate patients from the medical environment or treatment room and help to focus on pleasant stimuli so that it can reduce negative feelings such as anxiety [17].

This study was conducted to develop a nonpharmacological therapy by utilizing VR technology to reduce anxiety levels in cancer patients undergoing chemotherapy. The VR technology developed provides the experience of exploring blue space natural scenery in the Seribu Islands, Indonesia, virtually, so it is hoped that patients can be distracted from feelings of anxiety and that their anxiety levels can be reduced after using VR.

Given the ethical and practical constraints of immediate implementation in clinical settings, this research was designed as a preliminary study involving healthy adult participants. The purpose was to evaluate the feasibility, usability, and potential anxiety‐reducing effects of the VR Seribu Island environment prior to its application among cancer patients. Specifically, this study aimed to assess the effectiveness of the developed VR, evaluate user experiences (UXs) related to visual and auditory content, and identify the occurrence of cybersickness symptoms. In addition, changes in anxiety scores before and after VR exposure were analyzed. Findings from this exploratory phase will serve as foundational evidence for future clinical studies targeting cancer patients undergoing chemotherapy.

## 2. Methods

### 2.1. Study Design

This study employed a mixed‐methods design, combining both quantitative and qualitative approaches. It involved a single group of participants, with measurements taken before (pretest) and after (posttest) the use of the VR Seribu Island. Assessments were conducted on anxiety scores (pre‐ and post‐VR), cybersickness syndrome (post‐VR), and UX (post‐VR). A qualitative analysis was performed through semi‐structured interviews to gain deeper insights into users’ experiences with the VR application. This research was designed as a preliminary mixed‐methods pilot study aimed at assessing the feasibility, usability, and potential anxiety‐reducing effects of the VR Seribu Island program. The study comprised two phases: the development of the VR content and a feasibility test with a group of healthy individuals.

### 2.2. Instruments

We used the Meta Quest 2 as the VR device due to its ability to provide a highly immersive experience, supported by its comfortable design, powerful performance, and intuitive controls. This standalone device operates without the need for a computer or phone, though it requires pairing with a mobile app and connection to the same Wi‐Fi network. The Meta Quest 2 includes two touch controllers with hand‐tracking technology and features 6 GB of upgraded RAM, powered by the Qualcomm Snapdragon XR2 platform, ensuring smooth and responsive interactions. Additionally, its high‐resolution display of 1832 × 1920 pixels delivers sharp and clear VR visuals [18].

### 2.3. Participants

The research sample consisted of 30 healthy adults recruited through WhatsApp flyers and online announcements. Participants were selected using a convenience sampling approach based on voluntary participation and availability. Inclusion criteria include age 18–40 years, healthy, and not taking medication regularly due to diseases such as hypertension, epilepsy, and diabetes mellitus. Participants who used glasses, contact lenses, or other visual aids were excluded, as were those with visual, hearing, or sensory impairments or excessive fear of heights, water, or the ocean.

Although the Meta Quest 2 can accommodate prescription glasses using a spacer, participants who relied on glasses or other visual aids were excluded. This decision was made to ensure optimal visual clarity and minimize potential issues related to lens reflection, headset–face misalignment, reduced field of view, and variability in visual comfort, all of which could affect immersion and data accuracy. Similar exclusion criteria have been applied in previous VR‐based research to reduce sensory interference during immersive sessions [19]. This hardware‐related constraint may limit the generalizability of the findings.

At the time of the study, prescription lens inserts and headsets with adjustable diopter settings were not available within the experimental setup, necessitating the exclusion of participants who relied on visual corrections. Future studies could address this limitation by employing clip‐in prescription lens inserts or VR headsets with adjustable diopter mechanisms, which would allow the inclusion of participants wearing glasses while maintaining visual fidelity, comfort, and data reliability. Such approaches would enhance accessibility and improve the generalizability of findings across a broader population.

### 2.4. Measures

This study employed three instruments: the State–Trait Anxiety Inventory (STAI), the Cybersickness Syndrome Questionnaire (CSQ‐VR), and a UX questionnaire adapted from several previous studies. The State Anxiety subscale of the STAI [20] was used to assess participants’ current levels of anxiety. The STAI for adults is a proprietary instrument (Copyright 1968, 1977 by Charles D. Spielberger; published by Mind Garden, Inc., www.mindgarden.com), and a formal license to administer it was obtained from Mind Garden, Inc. The Indonesian version used in this study was adapted and validated by Haizatullah et al. [21], who reported all 20 items as valid with a Cronbach’s *α* of 0.893, indicating high internal consistency. The questionnaire consists of 20 items rated on a 4‐point Likert scale (1 = not feeling at all and 4 = feeling very much), with higher scores indicating greater anxiety.

The CSQ‐VR was used to assess cybersickness symptoms that may occur after VR exposure. The questionnaire consists of six items rated on a 7‐point Likert scale (1 = “absent feeling,” 2 = “very mild feeling,” 3 = “mild feeling,” 4 = “moderate feeling,” 5 = “intense feeling,” 6 = “very intense feeling,” and 7 = “extreme feeling”). The items cover three symptom categories: nausea, vestibular, and oculomotor. Total scores range from 6 to 42, with higher scores indicating greater VR‐related discomfort [22]. The CSQ‐VR was translated into Indonesian using a forward–backward translation procedure to ensure conceptual equivalence and cultural appropriateness. The translated version was tested for psychometric properties, showing that all items were valid (*r* > 0.361) and internally consistent (Cronbach’s *α* = 0.733). These findings indicate that the Indonesian CSQ‐VR is a valid and reliable tool for assessing cybersickness symptoms in this study population.

The UX questionnaire was adapted from several established instruments related to technology acceptance and user engagement [23–28]. The questionnaire consisted of 19 statement items rated on a 5‐point Likert scale (1 = strongly disagree to 5 = strongly agree) and was designed to assess seven subscales: perceived ease of use, perceived control, presence, satisfaction, attitude, enjoyment, and intention to use. Psychometric testing in the present study showed that all items were valid (*r*_observed > *r*_table = 0.361) and internally consistent, with a Cronbach’s *α* = 0.747, indicating acceptable reliability for exploratory research. In addition to the quantitative assessment, UX was further explored through semi‐structured interviews to obtain deeper insights into participants’ perceptions of the quality and immersion of the VR content.

Meanwhile, qualitative data were collected through semi‐ structured interviews conducted by the principal researcher. A semi‐structured interview is a qualitative research approach that involves a set of predetermined open‐ended questions designed to stimulate discussion while also allowing the interviewer to delve deeper into specific themes or responses as they arise [29]. The interviews were conducted right after the participants completed the UX questionnaire. The interviews were conducted using an interview guide containing open‐ended questions related to participants’ feelings after using VR, device comfort, the most liked and disliked video content and settings, along with the reasons and opinions on audio, visuals, and overall VR.

### 2.5. VR Seribu Island

**Figure 1:**
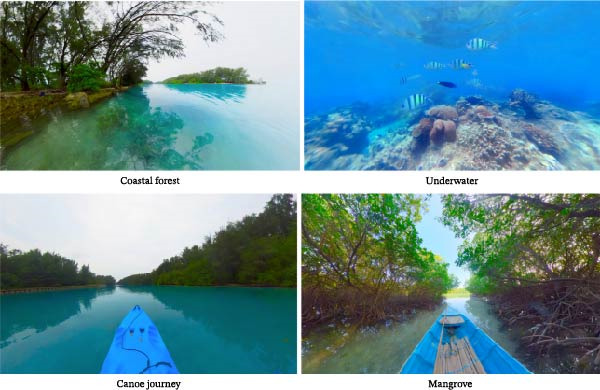
VR Seribu Island video content.

The VR Seribu Island application features 360° video scenes showcasing the landscapes of the Seribu Islands, Indonesia. The content comprises four immersive blue space scenes: an underwater view, a coastal forest, a canoe journey, and a trek through a mangrove forest (Figure 1). The video content includes both static scenes (coastal forest and underwater) and dynamic scenes (canoe journey and mangrove forest) that provide slight movements when viewed. Each scene is complemented by additional settings that can be toggled on or off, including background music, natural sounds (such as crashing waves, bird chirps, and wind gusts), and animations (such as bubbles and fireflies). The copyright of this program has been registered with the Ministry of Law and Human Rights of the Republic of Indonesia (Number EC00202448071).

### 2.6. VR Seribu Island Development Process

The VR Seribu Island content was developed through a systematic process. The development team consisted of researchers with backgrounds in medical education, psychiatry, and multimedia technology. The development of VR Seribu Island was informed by attention restoration theory and stress reduction theory, which suggest that exposure to natural environments can help restore attention and reduce stress. Blue space environments were selected based on evidence indicating the calming effects of water‐associated settings on psychological well‐being. The VR application was designed to serve as a distraction technique, redirecting the user’s attention away from anxious thoughts toward pleasant and immersive natural stimuli.

The VR content was created by capturing 360° videos at various locations in the Seribu Islands Regency, Indonesia. Video recording was conducted using a 360° camera (Insta360 ONE X2). Four distinct scenes were selected: a coastal forest, an underwater view featuring coral reefs and marine life, a canoe journey across open water, and a trek through a mangrove forest. Each scene had a duration of ~3–4 min, resulting in a total exposure time of about 15 min. The videos were processed to ensure smooth playback on the Meta Quest 2 headset.

Audio and visual elements were incorporated to enhance the immersion. Each scene was developed with three additional settings (natural sounds, background music, and animations), which could be toggled on or off independently, allowing participants to experience variations in sensory input. The natural sounds included elements such as crashing waves, bird chirps, and wind gusts, tailored to match each scene. The background music consisted of slow‐tempo instrumental tracks. Animations, such as bubbles in the underwater scene and fireflies in the coastal forest, were added to increase the realism and engagement.

Prior to the feasibility testing phase, the VR content was reviewed by the research team, which included a medical education specialist, a psychiatrist, and multimedia and VR technology experts. The review focused on content appropriateness, visual and audio quality, and the potential for cybersickness. Based on feedback from the review process, several refinements were made, including the reduction of rapid camera movements in dynamic scenes to minimize dizziness, the adjustment of audio volume levels to ensure balanced sensory input, and the addition of more colorful marine life to enhance visual appeal. The final version of the VR Seribu Island content was determined to be suitable for the feasibility testing phase.

### 2.7. Study Procedures

This study began with taking 360° videos at tourist locations in the Seribu Islands Regency to be used as VR content. Videos were recorded on‐site at several interesting places to create a more realistic virtual experience for users. We developed the 360° videos into single‐player VR content used in this study. After the VR program was developed, recruitment of research subjects began. Recruitment was done by distributing e‐flyers via WhatsApp messages in early May 2024. Participants who met the inclusion criteria were asked to fill out a registration form via the link available on the e‐flyer. The registration link was closed after reaching 30 people. Those willing to be our research subjects signed a written consent form (informed consent). Data collection was subsequently conducted over 2 days on 20–21 May 2024.

Before starting the experiment, the research team briefed each participant on the research procedures and demonstrated how to use the VR device. Participants had to fill out the STAI questionnaire as a measure of initial anxiety (pretest). Furthermore, participants were directed to a separate room to view VR content. The room was provided for one participant at a time to avoid interference from other participants. The research team helped participants put on the Meta Quest 2 device and made sure they were seated in the most comfortable position.

Participants then engaged with a 15‐min VR experience, consisting of four distinct videos: (a) a seaside forest, (b) underwater, (c) a canoe trip in the middle of the blue sea, and (d) a trip through a mangrove forest. Each video was presented with four different settings: (a) setting 1: music on, nature sounds on, and animations on, (b) setting 2: music off, nature sounds off, and animations on, (c) setting 3: music on, nature sounds on, and animations off, and (d) setting 4: music off, nature sounds off, and animations off.

After watching VR, participants filled out a post‐VR questionnaire, including anxiety, cybersickness syndrome, and UX questionnaires. After completing the questionnaire, participants proceeded to a small group interview session (2–3 people) led by the research team. One researcher guided the interview with open‐ended questions, while another recorded the interview session. After the interview was completed, the participants were allowed to leave the research location.

### 2.8. Statistical Analysis

We calculated descriptive statistics for the key demographics. The data were analyzed with the SPSS (Version 21.0, IBM Inc.). Respondent characteristic data including age, gender, education, health condition, previous VR experience, and type of device used, are presented in table form (frequency, min–max, mean, and standard deviation). Normality was assessed using the Shapiro–Wilk test, which indicated that the anxiety score data were not normally distributed (*p*  < 0.05). Therefore, the Wilcoxon Signed‐Rank test was used to compare pre‐ and post‐VR anxiety scores. The effect size (*r*) for the Wilcoxon Signed‐Rank test was calculated using the formula 𝑟 = 𝑍/√*N*, with values of 0.1, 0.3, and 0.5 interpreted as small, medium, and large effects, respectively. Median differences and 95% confidence intervals were obtained using the bias‐corrected and accelerated (BCa) bootstrap procedure.

Statistical analysis was performed on the total STAI anxiety score (sum of 20 items) to assess overall state anxiety before and after VR exposure. Item‐level data were presented descriptively (median and IQR) without inferential testing, as the analysis focused on the overall anxiety construct rather than individual items.

Given the exploratory and preliminary nature of this study, no a priori power calculation was performed. The sample size (*N* = 30) was determined based on feasibility and comparability with similar pilot studies evaluating VR‐based relaxation interventions. Therefore, the findings should be interpreted as indicative rather than confirmatory, serving primarily to inform future studies with larger and more diverse samples.

### 2.9. Qualitative Analysis

We conducted semi‐structured cognitive debriefing interviews with participants after exposure to VR, asking questions about their overall experience, the VR device, and the software, including asking for VR video content and the most likeable and the least VR video settings.

Qualitative data from semi‐structured interviews were analyzed using reflexive thematic analysis following Braun and Clarke’s [30] six‐phase framework. Two researchers independently read and coded the transcripts to familiarize themselves with the data and generate initial codes. They then met to discuss their interpretations and reach consensus on the emerging themes. Coding was conducted inductively to allow themes to arise from the data rather than being predetermined. Thematic patterns were reviewed and refined through iterative discussion to ensure coherence and consistency. Interrater reliability was maintained through ongoing comparison and agreement on coding decisions. The final themes were selected based on their relevance to the study objectives and represent participants’ shared experiences of the VR Seribu Island.

### 2.10. Ethics and Informed Consent

This study was approved by the Ethics Committee of the Faculty of Medicine, Universitas Indonesia (KET‐1613/UN2.F1/ETIK/PPM.00.02/2023). Participants provided their consent to the study’s terms through an informed consent form, which detailed the study’s purpose and benefits, the research procedures, and data confidentiality. The participants independently completed and signed the informed consent form.

## 3. Results

Thirty participants were involved in this study. The mean age of participants was 30.97 ± 5.53 years, with ages ranging from 23 to 40 years. Most participants were female (60.0%), and most had a bachelor’s degree (63.3%). Only eight participants (26.7%) reported prior experience with VR, with the most used device being the Oculus Quest (62.5%) (Table 1).

**Table 1:** Participant demographics.

Characteristics	Mean ± SD	Min–max	*N* (%)
Age (years)	30.97 ± 5.53	23–40	—
Gender			
Male	—	—	12 (40.0)
Female	—	—	18 (60.0)

Education background			
Senior high school	—	—	3 (10.0)
Associate degree	—	—	2 (6.7)
Bachelor’s degree	—	—	19 (63.3)
Master’s degree	—	—	6 (20.0)

Experience with VR			
Yes	—	—	8 (26.7)
No	—	—	22 (73.3)

Frequency of prior VR use (times)	0.53 ± 1.04	0–4	—

Type of device			
Oculus Quest	—	—	5 (62.5)
VR box	—	—	3 (37.5)

### 3.1. UX of VR

The UX for VR had an average score of 4.48 (SD = 0.34) out of 5 points. The top two scoring items in the UX questionnaire were “I am really delighted with the virtual experience” (4.80) and “I really enjoyed this VR experience” (4.70). The two lowest scoring items were “I felt like the objects in the presentation surrounded me” (4.00) and “It was as though my true location had shifted into the VR environment” (4.13) (Table 2).

**Table 2:** User experience (*N* = 30).

Subscale	Items	Min–max	Mean ± SD
Perceived ease of use (PEU)	1. I have found it easy to use VR (PEU1)	3–5	4.43 ± 0.57
	2. Learning how to use VR was easy (PEU2)	4–5	4.57 ± 0.50

Perceived of control (PC)	3. I felt I could deal with the VR device (headset, controller) and control them easily (PC1)	3–5	4.47 ± 0.63
	4. I felt that in the virtual environment, I could control my movements (PC2)	3–5	4.40 ± 0.62

Presence (P)	5. I felt like I was actually there in the VR environment (P1)	3–5	4.43 ± 0.73
	6. It was as though my true location had shifted into the VR environment (P2)	1–5	4.13 ± 1.09
	7. I felt like I could move around among the objects of VR (P3)	3–5	4.27 ± 0.58
	8. I felt like the objects in the presentation surrounded me (P4)	2–5	4.00 ± 0.79

Satisfaction (S)	9. My interaction with VR is really satisfying (S1)	4–5	4.44 ± 0.50
	10. I am really delighted with the virtual experience (S2)	4–5	4.80 ± 0.41
	11. The virtual experience has worked as well as I expected it would (S3)	3–5	4.30 ± 0.60

Attitude (A)	12. I like the concept of exploring natural landscapes through virtual reality (A1)	3–5	4.57 ± 0.57
	13. Using this VR is a worthy decision (A2)	4–5	4.70 ± 0.47

Enjoyment (E)	14. I really enjoyed this VR experience (E1)	4–5	4.77 ± 0.43
	15. The experience is very exciting (E2)	4–5	4.47 ± 0.47
	16. The experience made me happy (E3)	3–5	4.40 ± 0.62
	17. The experience makes me feel like I really achieved something new (E4)	4–5	4.60 ± 0.50

Intention to use (IU)	18. When using VR, I felt like I could actually see myself visiting natural landscapes (IU1)	3–5	4.50 ± 0.57
	19. After using VR, it is like that I will visit Kepulauan Seribu in the future (IU2)	3–5	4.57 ± 0.57

**Figure 2:**
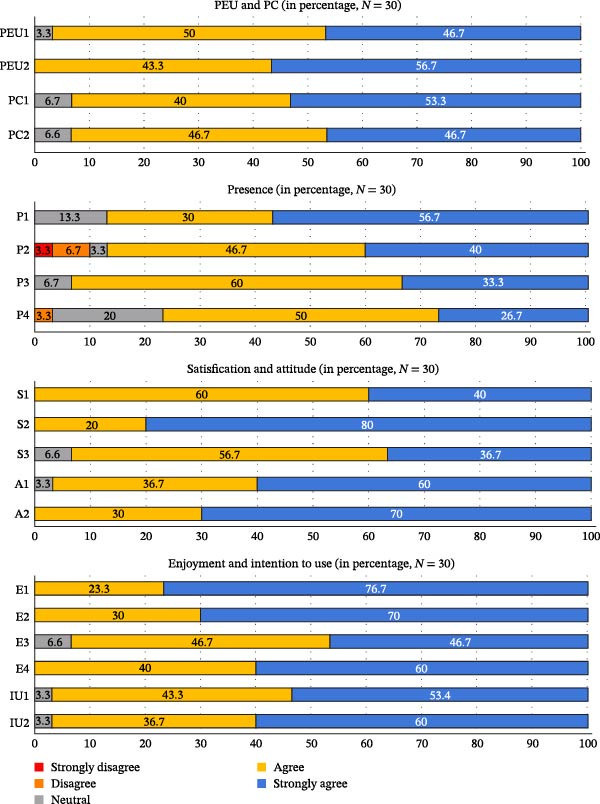
User experience of VR.

Figure 2 presents the percentage of participants who agreed across all subscales of UX was an average of 94.9%. Participants reported a very high level of perceived ease of use, with 100% agreeing (strongly agree and agree) that they found it easy to learn how to use VR. Additionally, the highest level of satisfaction was recorded, with 100% agreeing (strongly agree and agree) that their interaction with VR was very satisfying, and 100% expressed that they were very delighted with the virtual experience. Regarding attitudes, 100% of participants agreed (strongly agree and agree) that using VR was a worthwhile decision. Finally, a high level of enjoyment was also reported, with 100% of participants agreeing (strongly agree and agree) that they thoroughly enjoyed the VR experience, 100% feeling that the experience was very exciting, and 100% feeling that they had achieved something new through the VR experience.

**Figure 3:**
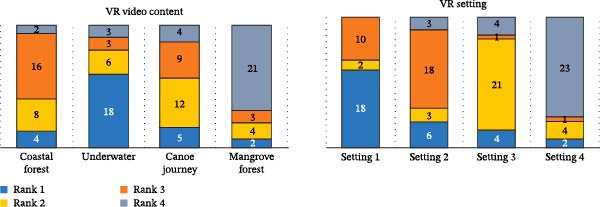
Participant preferences of VR video content and setting.

Participants also ranked the VR video content (coastal forest, underwater, canoe journey, and mangrove forest) and VR video settings (music, natural sounds, and animations), with rank 1 being the most preferred and rank 4 being the least preferred. As presented in Figure 3, “underwater“ was the most favored VR content, while “setting 1 (music on, nature sound on, and animation on)” was the most preferred video setting among participants. Conversely, “mangrove forest” and “setting 4 (music off, nature sound off, and animation off )” were the least favored.

### 3.2. Cybersickness Syndrome

The mean scores for subscales of CSQ‐VR, including nausea A (nausea), nausea B (dizziness), vestibular A (disorientation), vestibular B (postural instability), oculomotor A (visual fatigue), and oculomotor B (visual discomfort), were relatively low, ranging from 1.27 to 1.73 on a scale of 1–7, where 1 indicates the absence of symptoms and 7 indicates extreme symptoms. The standard deviations, ranging from 0.64 to 1.11, suggest moderate variability among participants, with most experiencing mild symptoms (Table 3).

**Table 3:** Cybersickness syndrome (CSQ–VR) scores.

No	Items	Min–max	Mean ± SD
1	Nausea A: do you experience nausea (e.g., stomach pain, acid reflux, or tension to vomit)?	1–6	1.37 ± 1.10
2	Nausea B: do you experience dizziness (e.g., light‐headedness or spinning feeling)?	1–5	1.67 ± 1.09
3	Vestibular A: do you experience disorientation (e.g., spatial confusion or vertigo)?	1–3	1.27 ± 0.64
4	Vestibular B: do you experience postural instability (i.e., imbalance)?	1–4	1.60 ± 0.97
5	Oculomotor A: do you experience a visually induced fatigue (e.g., feeling of tiredness or sleepiness)?	1–4	1.57 ± 0.94
6	Oculomotor B: do you experience a visually induced discomfort (e.g., eyestrain, blurred vision, or headache)?	1–5	1.73 ± 1.11
7	CSQ–VR total	6–20	9.20 ± 3.47

**Figure 4:**
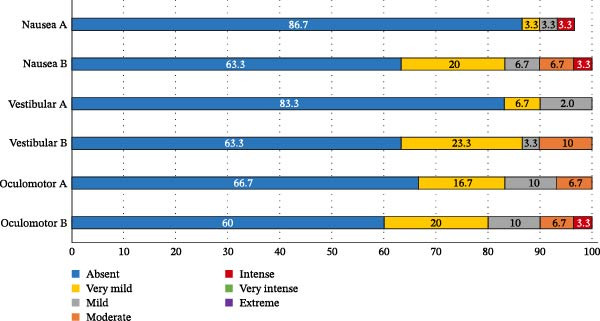
Distribution of cybersickness symptom severity among participants 349 across six CSQ‐VR items (in percentage).

Figure 4 illustrates the distribution of cybersickness symptom severity across six CSQ‐VR items. A significant majority of participants reported no symptoms (absent feeling) for most items, with “nausea A” and “vestibular A” showing the highest absence rates at 86.7% and 83.3%, respectively. While intense had appeared in a small percentage of cases (<5%) in “nausea A,” “nausea B,” and “oculomotor B.”

### 3.3. Anxiety Scores Pre and Post Using VR

**Figure 5:**
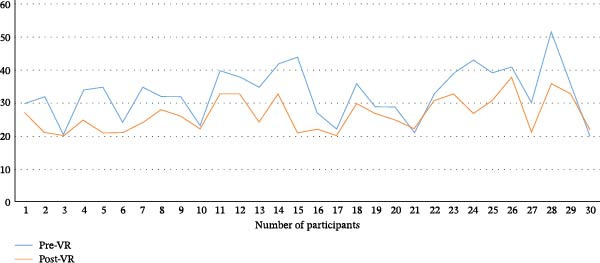
Comparison of individual anxiety scores (STAI) before and after exposure to the VR Seribu Island (*N* = 30). Each line represents one participant’s pre‐ and post‐VR scores.

The STAI‐State (state anxiety) questionnaire was completed by respondents before and after using VR. Figure 5 presents a comparison of anxiety scores before and after exposure to the VR Seribu Island experience for 30 participants. The data reveal a noticeable reduction in anxiety levels post‐VR exposure for most participants. The pre‐VR anxiety scores (blue line) fluctuate at higher levels compared to the post‐VR scores (orange line). The median (IQR) anxiety scores decreased from 33.5 (11.0) before VR to 25.5 (10.0) after VR, confirming a significant reduction in anxiety (*p*  < 0.001, *r* = 0.83).

Inferential analysis was conducted on the total STAI‐State score to evaluate the overall state anxiety reduction following the VR Seribu Island experience. State anxiety scores significantly decreased following VR exposure, with the median score reducing from 33.5 (IQR = 11.0) before VR to 25.5 (IQR = 10.0) after VR. The median difference was 8.0 (95% CI [4.39, 8.61], *p* = 0.001), with a large effect size (*r* = 0.83). These results confirm a significant reduction in state anxiety levels after the intervention.

### 3.4. Qualitative Results

Three themes emerged from their experiences of the VR Seribu Island: “sense of realism and presence,” “emotional and relaxing experience,” and “Minor Discomfort and Initial Dizziness.” Furthermore, this Section 3.4.4 explores specific participant preferences regarding the VR video content and settings.

#### 3.4.1. Sense of Realism and Presence

Many participants reported feeling as though they were truly present in the locations depicted in the VR experience. They described the experience as “realistic,” “feels like being there,” and “as if present at the scene.” Some even mentioned initial disorientation, which suggests a high level of immersion.

*“Feels like actually being at the location in the video, very real…”* (P12)
*“Exciting, feels like being there for real….”* (P16)
*“Pleasant, feels like being inside the world, sounds seem authentic”* (P18)
“*Feels realistic*, *as if among the contents”* (P20)
*“I felt calm, almost as if I were actually present at the scene being watched”* (P27)
*“Refreshing and healing, similar to real tourism experiences…”* (P29)

#### 3.4.2. Emotional and Relaxing Experience

Numerous participants expressed feelings of happiness, comfort, and relaxation after using VR. Words like “happy,” “relaxing,” “comfortable,” and “calm” were frequently mentioned. The VR experience was also associated with positive emotions, such as the desire to go on a vacation or explore new places.

*“I feel calm and peaceful. I enjoy the moment”* (P4)
*“I feels so happy and relax”* (P6)
*“Make me more relax and comfortable”* (P9)
*“Enjoyable*, *relaxing*, *with sound making it more melancholic*, *animations adding to the*
*mood”* (P17)
*“Reminiscent of holidays*, *having been to the Seribu Islands helps me visualize and*
*relate*, *making it comfortable and relaxing”* (P28)

#### 3.4.3. Minor Discomfort and Initial Dizziness

While most participants felt comfortable, some experienced slight discomfort or dizziness, particularly at the beginning of the experience or during scenes with dynamic movement. However, this sensation generally diminished as they became accustomed to the VR environment.

“*Slightly dizzy during dynamic movements*, *especially on the blue boat/canoe in the middle of the ocean*, *similar to the real-life experience of feeling dizzy in such situations”* (P22)
*“I felt slightly dizzy at the beginning, but once I became immersed in the environment, the dizziness went away”* (P23)
*“I felt a bit dizzy during the dynamic scenes in the video, as if I were moving along with the motions. I then became slightly disoriented because the sensation of sitting in the boat differed from actually sitting on a bench”* (P28)

#### 3.4.4. Participant Preferences on VR Video Content and Settings

Based on Figure 2, participants expressed a strong preference for the underwater VR video content due to its vibrant and clean visuals, including colorful fish and coral reefs. Many participants found it exciting because it offered a rare and realistic experience of being underwater, which they had never encountered before.

*“When all video settings are on*, *it feels harmonious and fitting*, *with the presence of fish*
*making it not boring”* (P8)
*“I had never seen coral reefs in person before, only in pictures, but watching the VR video felt like really seeing them and it was a new experience. I liked the underwater scene the most because it was the most colorful”* (P26)
*“I like swimming, so I enjoyed seeing the colorful fish. The animation and music made it more relaxing”* (P27)
*“The coral is beautiful, the fish feel real, and the ASMR effect of the water sounds ’gulp gulp’ is very realistic”* (P29)

While setting 1 (music on, nature sound on, and animation on) was the most preferred video setting among participants. They appreciated the natural sounds and bubble animations, which added a sense of realism and vitality to the scenes, particularly in the underwater videos. The combination of music and nature sounds created a calming and soothing environment, while the animations enhanced the overall experience by making it feel more vibrant and alive.

*“I feels relax the most with this setting”* (P12)
*“Most enjoyable, calming, nature sounds are soothing”* (P14)
*“The music and sounds enhance the scenery, making it feel more realistic, and the animations feel realistic”* (P24)
*“Complete package, all stimuli received, visuals and audio are perfect. Between the effects of music and natural sounds, I prefer natural sounds because they feel more real, but the music does not really bother me”* (P29)

## 4. Discussion

This study provided preliminary insights into the effectiveness and UX of the VR Seribu Island using data from 30 healthy participants. The current study demonstrates that anxiety levels were significantly reduced following exposure to VR. The median anxiety score decreased from 33.5 (pre‐VR) to 25.5 (post‐VR), with a median difference of 8.0 (95% CI [4.39, 8.61], *p* = 0.001, *r* = 0.83), indicating a large effect size. This suggests that the VR environment, showcasing natural scenery, can serve as an effective tool to reduce anxiety. Although the effect size was large, these findings should be interpreted cautiously due to the small sample size and the absence of a control group. The lack of a control group limits the ability to attribute the reduction in anxiety solely to the VR intervention as other factors such as the passage of time, relaxation, or novelty effects may have contributed to the observed changes. Future research should incorporate a controlled experimental design to differentiate the specific contribution of the VR experience from these potential confounding factors.

The findings align with a growing body of evidence that supports the efficacy of VR‐based interventions for reducing anxiety across various populations. For instance, Chirico et al. [14] found that VR combined with music therapy significantly reduced anxiety in breast cancer patients undergoing chemotherapy. Similarly, Indovina et al. [16] reported reduced pain and distress during medical procedures using VR distraction techniques. However, in contrast to these clinical studies, which focused on patients in healthcare settings, this study utilized health participants. While in both patients and healthy participants, the studies showed a reduction in anxiety, the degree of anxiety reduction may differ when used in a clinical population that typically experiences higher baseline anxiety levels due to medical treatment. Moreover, while “VR Seribu Island” used natural landscapes as its core content, which participants found calming, other studies often used game‐based or abstract VR environments. For example, a study by Sharar et al. [17] used a VR game in a hospital setting, which also led to reduced anxiety and pain, but the immersive game format differs from the nature‐based distraction method used in this study. This suggests that different types of VR environments, whether nature‐based or game‐based, can achieve similar outcomes, but the user’s subjective experience may vary.

Recent systematic reviews also reinforce the potential of VR as a supportive tool for oncology patients undergoing chemotherapy. Gautama et al. [31] demonstrated through a meta‐analysis of randomized controlled trials that immersive VR effectively reduces anxiety, depression, fatigue, pain, and physiological stress in cancer patients, highlighting its multidimensional therapeutic benefits. Likewise, Alvarado‐Omenat et al. [32] confirmed that VR interventions significantly reduced anxiety, pain, and stress in chemotherapy patients, though they emphasized the need for standardized treatment protocols and more rigorous randomization procedures. These findings strengthen the translational relevance of the present preliminary study and suggest that nature‐based immersive environments, such as the VR Seribu Island, could offer similar psychological benefits when adapted for clinical oncology settings.

Participants also reported a highly positive UX, with an overall satisfaction score averaging 4.48 out of 5. They particularly enjoyed the visual and audio elements, with high satisfaction (4.80) and enjoyment (4.70) scores. High levels of enjoyment and ease of use were also noted, which reinforces the potential of VR as a therapeutic tool. Ease of use was also rated positively, with 100% of participants agreeing that the VR device was easy to use. Among the four VR scenes (underwater, coastal forest, canoe journey, and mangrove forest), participants preferred the underwater scene the most. The combination of music, nature sounds, and animations (Setting 1) was the most favored, enhancing the sense of immersion and relaxation. Their liking for the sound setting was related to their satisfaction with the audio elements.

The VR content, set in the Seribu Islands, resonated with participants, helping them feel emotionally connected and calm. Many reported a sense of realism and presence in the virtual environment, which likely contributed to the reduction in anxiety. Most participants expressed positive emotions like calmness, relaxation, and happiness, aligning with the intended purpose of VR as a distraction technique to alleviate anxiety.

Regarding cybersickness, most participants experienced minimal symptoms, with mean scores ranging between 1.27 and 1.73 on a 7‐point scale, suggesting that the VR content was well‐received and did not cause significant discomfort.

One notable strength of this study is its focus on the UX. By combining quantitative and qualitative data, the study not only measured the reduction in anxiety but also provided deep insights into how participants perceived the VR environment’s usability, immersion, and cybersickness symptoms. This holistic approach is similar to studies like Davis et al. [23], which also used a user‐centered design approach to VR intervention testing, offering both experiential and efficacy feedback.

This study shares several common limitations with other VR‐based studies, including its small sample size (*N* = 30) and the short duration of exposure. While the results were significant, larger sample sizes are necessary to confirm the findings and allow for more nuanced analysis of user subgroups. Similarly, studies by Chirico et al. [14] and Indovina et al. [16] used small samples and short‐term exposure. These factors limit the ability to generalize the results to broader or more diverse populations.

Furthermore, the age range of participants in this study was restricted to healthy adults aged 23–40 years. This narrow age range limits the generalizability of our findings, particularly to elderly patients, who may be the primary target population for VR‐based anxiety interventions in clinical oncology settings. Older adults may have different levels of familiarity with VR technology, varying susceptibility to cybersickness, and distinct perceptual and cognitive responses to immersive environments. Therefore, the observed anxiety‐reducing effects may not directly translate to older populations. Future studies should include participants across a wider age spectrum, including elderly individuals, to assess the applicability of VR Seribu Island in diverse age groups.

Another limitation is that this study used healthy participants, whereas many VR studies focus on clinical populations. This limits the generalizability to individuals with higher baseline anxiety or other health conditions. In contrast, the studies involving cancer patients or individuals undergoing medical procedures are more applicable to clinical settings, offering a more direct understanding of VR’s impact in healthcare.

Additionally, while this study monitored cybersickness symptoms, it did not examine the long‐term use of VR. Some studies, like Kourtesis et al. [22], specifically focus on validating questionnaires for measuring cybersickness over prolonged VR sessions, addressing concerns about the possible increase in discomfort with repeated use. Long‐term effects, both in terms of anxiety reduction and side effects, remain unexplored in this study.

In comparison to studies focused solely on clinical outcomes, such as anxiety or pain reduction, this study’s user‐experience insights provide valuable information for optimizing the design of future VR applications. For instance, knowing that participants rated ease of use and satisfaction highly allows developers to create content that minimizes the cognitive load and enhances accessibility. A recent study by Sun et al. [33] also shows that green spaces in VR simulations effectively reduce anxiety levels in users. Meanwhile, blue spaces have the potential to ease the mind and emotions [15]. These immersive environments provided by the natural scenery will be beneficial for those with anxiety.

In this study, the observed user preferred music and nature sounds over animation within environments. These ambient audio elements can help create a more immersive and calming experience, which is particularly important for individuals seeking to reduce anxiety through VR simulation [34, 35]. The soothing effects of natural soundscapes and nonintrusive background music have been shown to have a positive impact on stress and emotional regulation [36, 37]. In contrast, overly animated or distracting visual elements may be counterproductive as they can be visually overwhelming and divert attention from the intended therapeutic or training objectives. By prioritizing audio cues that mimic real‐world environments, the VR Seribu Island can foster a sense of presence and familiarity that enhances the effectiveness of the simulation.

Moreover, the Seribu Island VR content’s cultural relevance to participants, offering familiar or desirable landscapes, is another strength not often seen in similar studies. Other studies may use more generic or foreign VR environments, which can limit user engagement or emotional connection. The personal connection to the content in this study may have contributed to its anxiety‐reducing effect.

## 5. Conclusions

This study provides preliminary evidence that the VR Seribu Island, which features blue space environments such as coastal and underwater scenes, has the potential to reduce anxiety and provide a positive UX. The significant reduction in anxiety scores observed after VR exposure (median difference = 8.0, 95% CI [4.39, 8.61], *p*  < 0.001, *r* = 0.83) indicates that immersive nature‐based VR may serve as an effective nonpharmacological approach for anxiety management. However, these findings should be interpreted with caution due to the small sample size and the absence of a control group. As a preliminary exploratory study involving healthy adults, the results are indicative rather than confirmatory. Future research with larger samples and clinical populations is needed to validate the therapeutic potential of blue space‐based VR, particularly among cancer patients undergoing chemotherapy.

## Funding

This research was funded by Universitas Indonesia (Grant NKB‐351/UN2.RST/HKP.05.00/2023).

## Conflicts of Interest

The authors declare no conflicts of interest.

## Data Availability

The data that support the findings of this study are available upon request from the corresponding author. The data are not publicly available due to privacy or ethical restrictions.
